# Phytobezoar—An Unusual Cause of Small Bowel Obstruction in Pediatric Age Group: A Case Report and Literature Review

**DOI:** 10.1002/ccr3.71809

**Published:** 2026-01-02

**Authors:** Aakash Pandit, Melisha Koirala, Aashish Panta, Nawadip Kandel

**Affiliations:** ^1^ Chitwan Medical College Teaching Hospital Bharatpur Chitwan Nepal; ^2^ National Medical College Teaching Hospital Birgunj Parsa Nepal; ^3^ University of Latvia Riga Latvia

**Keywords:** bezoar, case report, literature review, pediatric age group, phytobezoar, small bowel obstruction

## Abstract

Phytobezoars are the most common and well known type of bezoars yet one of the uncommon causes of mechanical obstruction of the small intestine. The reported prevalence rate of phytobezoars is estimated to be 0.4% despite being the 5th most common cause of acute small bowel obstruction. A previously healthy five‐year‐old girl presented to the Emergency Medicine Department with a 5‐day history of nonprojectile, nonbile stained vomiting, abdominal pain, and decreased urine output. Physical examinations revealed severe dehydration symptoms, and laboratory tests indicated abnormal electrolyte levels and metabolic alkalosis. The patient experienced a seizure, received medical interventions, and was diagnosed with mechanical intestinal obstruction due to a phytobezoar. After stabilization, she underwent surgical removal of the phytobezoar without complications and followed postoperative advice successfully. In cases without significant complications, surgical or aggressive medical treatment for bezoars may be unnecessary. Coca‐Cola, alone or combined with endoscopic methods, is effective in dissolving gastric phytobezoars, with success rates exceeding 90%. Conservative management involves proteolytic enzymes, cellulase, carbonated beverages, and endoscopic fragmentation. Clinicians should stay vigilant, as small bowel obstruction can occur up to 6 weeks later. Prokinetic agents and dietary guidelines help minimize bezoar formation. Surgical intervention, unlikely to address the root cause and potentially worsening motility issues, requires careful consideration. Phytobezoars are a significant consideration in pediatric small bowel obstruction cases. Conducting a thorough dietary history, focusing on fiber‐rich foods, is crucial. Radiographic and endoscopic studies aid in locating the phytobezoar. Timely surgical intervention is essential to prevent complications associated with small bowel obstruction.

## Key Clinical Message

Phytobezoars, though rare in children, should be considered in cases of unexplained intestinal obstruction, especially with a history of ingesting fibrous plant material. Early recognition through dietary history, imaging, and endoscopy enables timely intervention, preventing life‐threatening complications and unnecessary delays in management.

## 1 Introduction

Phytobezoars are the concretions of undigested plant materials. Studies have shown the prevalence of bezoars to be 0.4% [1]. Although rarely encountered, they remain the fifth most common cause of intestinal obstruction [2]. Typically discovered in the stomach in 78% of cases, these bezoars may alternatively manifest in the small intestine, accounting for up to 17% of occurrences. Phytobezoars, constituting around 40% of reported bezoar cases, primarily consist of indigestible vegetable fibers sourced from pulpy fruits, orange pits, seeds, roots, or leaves [3].

We report the case of a previously healthy 5‐year‐old girl who presented with intractable vomiting, abdominal pain, and decreased urine output, later found to have hypochloremic hypokalemic metabolic alkalosis and signs of sepsis. Further imaging and endoscopy revealed a phytobezoar lodged in the duodenum causing mechanical intestinal obstruction, necessitating surgical intervention. To our knowledge, such a presentation is rarely reported in this age group.

## 2 Case History/Examination

A previously healthy 5‐year‐old girl presented to the Department of Emergency Medicine with a 5‐day history of vomiting which was nonprojectile, nonbile stained, and nonblood stained and associated with intolerance of gastric feeding, a 5‐day history of abdominal pain which had a sudden onset, continuous course, nonradiating, and relieved with vomiting, and a 3‐day history of decreased urine output. The child had a history of frequently consuming persimmons along with plant leaves and fibrous stems, often inadequately chewed. There was no history of significant medical or surgical interventions, allergies, or inheritable conditions in the family. Physical examinations were remarkable for signs of severe dehydration, specifically an apathetic & lethargic appearance, dry oral mucosa, sunken eyes, and tachycardia. Crepitations were heard over the thorax upon auscultation.

## 3 Methods (Investigations and Treatment)

Her laboratory tests showed hemoglobin (Hb) (12.6 g%), white cell count (WBC) (18,210 per mm^3^) (neutrophils 75%, lymphocyte 15%), platelet count (729,000 per mm^3^), serum sodium (132 mmol/L), serum potassium (2.63 mmol/L), C‐Reactive Protein (2.6 mg/dL). The blood culture showed no growth after 72 h. Ultrasonography showed trace ascites and mild pleural effusion. Arterial blood gas analysis showed hypochloremic hypokalemic metabolic alkalosis with other dyselectrolytemia ↑p^H^ (7.64), ↑pCO_2_ (55.8 mmHg), ↓pO_2_ (49 mmHg), ↓ [Na^+^] (119 mmol/L), ↓ [K^+^] (2.37 mmol/L), ↓ [Ca^++^] (0.64 mmol/L), ↓ [Cl^−^] (59 mmol/L) (Table 1).

**TABLE 1:** Day‐wise clinical course, investigations, and management of the patient.

Day of admission	Presentation	Diagnostic testing	Interventions
1st day	Vomiting for 5 days Intolerance of gastric feeding for 5 days Abdominal Pain for 5 days Decreased urine output for 3 days Generalized tonic clonic seizure for 30 s Pediatric ICU admission	Hemoglobin: 12.6 g% TLC: 18,210 per mm^3^ DLC: Neutrophils 75%, lymphocyte 15% Platelet count 729,000 per mm^3^ Serum sodium: 132 mmol/L Serum potassium: 2.63 mmol/L C‐Reactive protein: 2.6 mg/dL Blood culture: no growth USG: trace ascites and mild pleural effusion ABG: hypochloremic hypokalemic metabolic alkalosis ↑p^H^: 7.64 ↑pCO_2_: 55.8 mmHg ↓pO_2_: 49 mmHg ↓ [Na^+^]: 119 mmol/L ↓ [K^+^]: 2.37 mmol/L ↓ [Ca^++^]: 0.64 mmol/L ↓ [Cl^−^]: 59 mmol/L USG Abdominal Scan	IV ondansetron 2 mg stat IV NS 10 mL/kg over 20 min IV Midazolam 1 mg stat WHO Plan C for Dehydration nCPAP 6 cm of H_2_O IV KCl Empirical antimicrobial therapy Seizure prophylaxis with Levetiracetam
2nd day	2 episodes of bilious vomiting	CECT abdomen and pelvis: suspected bezoar at D2/D3 junction	
3rd day	Fever spike 104°F at 1 PM	Esophagogastroduodenoscopy: confirmed bezoar at D2/D3 junction	IV Paracetamol 200 mg stat
4th day	Afebrile and hemodynamically stable		Transferred out of PICU to pediatrics ward
5th day			Surgical removal of bezoar

Abbreviations: ABG, arterial blood gas; CECT, contrast‐enhanced computed tomography; CRP, C‐reactive protein; D2/D3, second/third part of duodenum; DLC, differential leukocyte count; ICU, intensive care unit; IV, intravenous; KCl, potassium chloride; nCPAP, nasal continuous positive airway pressure; NS, normal saline; PICU, pediatric intensive care unit; TLC, total leukocyte count; USG, ultrasonography; WHO, World Health Organization.

**FIGURE 1:**
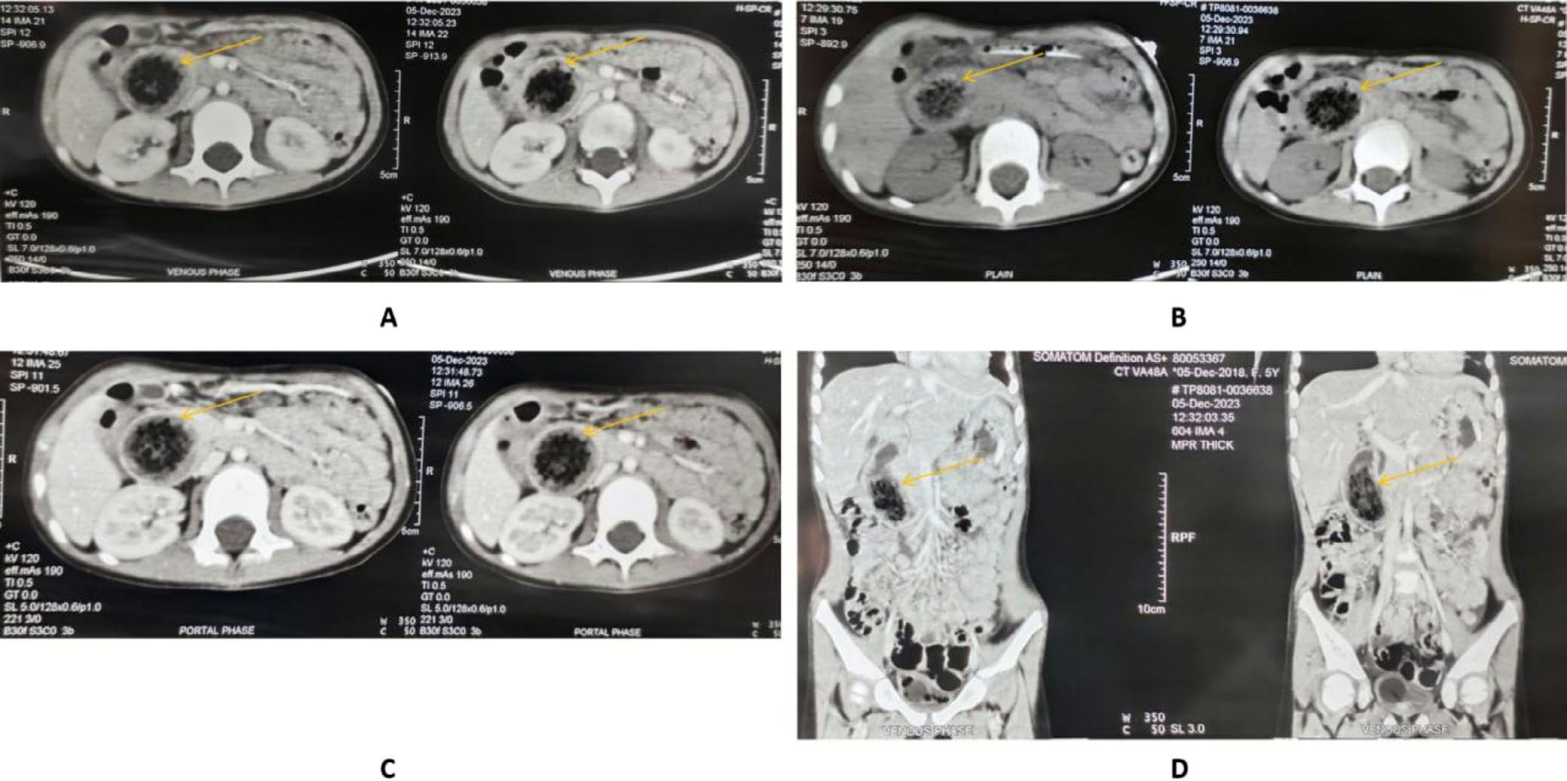
Computed tomography of abdomen showing phytobezoar (arrow shows phytobezoar). (A) Contrast enhanced axial section. (B) Plain axial section. (C) Contrast enhanced axial section. (D) Contrast enhanced coronal section.

**FIGURE 2:**
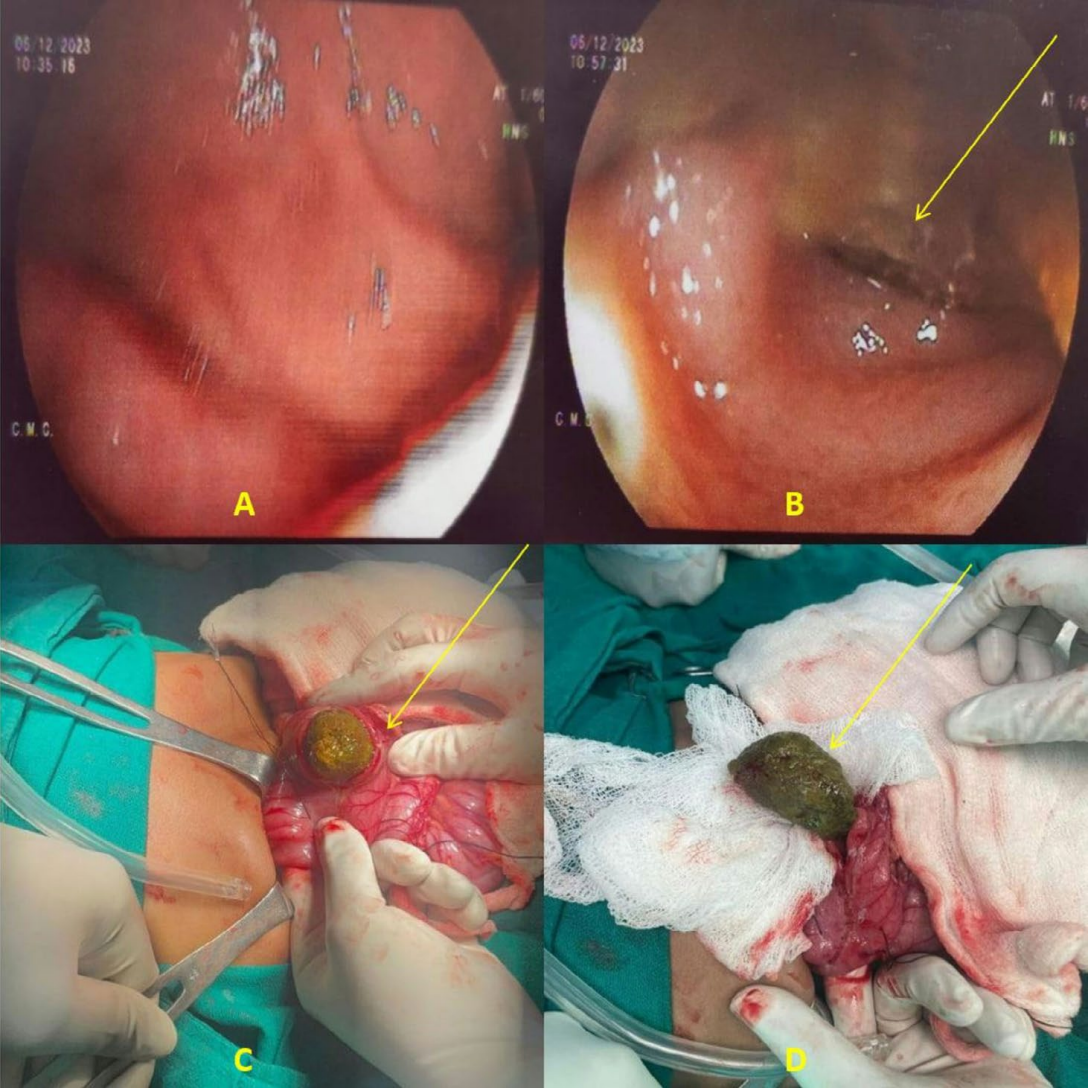
Images showing phytobezoar. (A) Endoscopic view of the first part of duodenum. (B) Endoscopic view of the second part of duodenum (arrow shows phytobezoar). (C) Surgical removal under process (arrow shows phytobezoar). (D) Surgical removal completed (arrow shows phytobezoar).

The child was treated with intravenous administration of 2 mg Ondansetron given stat and 10 mL/kg of normal saline over 20 min. Generalized tonic clonic seizure for 30 s followed by uprolling of eyes and clenching of teeth was observed, consistent with an acute symptomatic convulsion likely precipitated by profound electrolyte imbalance, for which 1 mg Midazolam was given intravenously stat. She was transferred to pediatric ICU where nCPAP 6 cm of H_2_O was provided and the correction of dehydration was continued according to WHO plan C but had to be discontinued subsequently due to suspected fluid overload as pertained by crepitations over lung field on auscultation. Correction and maintenance for hypokalemia was done. Empirical antimicrobial therapy was started for positive sepsis screening pertained by elevated CRP. Vancomycin was added to the regimen for suspected meningitis. Levetiracetam was started for the prophylaxis of seizure and CT Head revealed no meningeal enhancement. On the 2nd day of admission, two episodes of vomiting with bilious content were seen. CECT of Abdomen and Pelvis revealed distended D2 segment of duodenum with intraluminal mottled nonenhancing hypodense content and abrupt collapse of distal segment of duodenum, mild ascites, collapse consolidation of basal segments of left lower lobe and subsegmental atelectasis of lingula segment of left upper lobe (Figure 1). Possibility of bezoar lodged at D2 duodenum was suspected and further workup with esophagogastroduodenoscopy confirmed dilated D2 with bolus of impacted food at D2/D3 junction beyond which the scope couldn't be negotiated suggesting intestinal obstruction (Figure 2). Hence, diagnosis of mechanical intestinal obstruction due to phytobezoar was established. The patient was transferred out of PICU to pediatrics ward after she was hemodynamically stable, alert and active with no recurrence of seizure, cessation of vomiting for 58 h with normal passage of stool and urine output of 0.6 mL/kg/h. She was scheduled for the surgical removal of phytobezoar (Figure 2) (Table 1).

## 4 Clinical Course and Results (Outcome and Follow‐Up)

There were no postoperative complications and she adhered to and tolerated the advice of avoiding strenuous activities.

## 5 Discussion

Severe hyponatremia, as evidenced by a markedly reduced serum sodium level, was considered a probable precipitating factor for the seizure episode alongside the underlying sepsis‐related metabolic derangements.

In cases where there is no gastric outlet or small bowel obstruction or serious issues from bezoars such as perforation or uncontrollable hemorrhage, surgical or aggressive medical treatment may not be necessary. Coca‐Cola alone is effective in dissolving gastric phytobezoars in 50% of cases, and when combined with additional endoscopic methods, success rates exceed 90%. Conservative management of phytobezoars involves proteolytic enzymes, cellulase, carbonated beverages, and endoscopic fragmentation. However, clinicians must remain vigilant, as even after apparent dissolution, small bowel obstruction can occur up to 6 weeks later due to residual fragments. Prokinetic agents may be considered for patients at risk and dietary guidelines should be followed to minimize bezoar formation in such individuals. Given that surgery is unlikely to address the root cause of bezoar formation and could exacerbate gastric motility and emptying issues, careful deliberation is essential before opting for this intervention [1, 4, 5].

## 6 Conclusion

Phytobezoars are an important differential diagnosis of small bowel obstruction in the pediatric population. It is imperative to conduct a thorough dietary history assessment when addressing a case of small bowel obstruction, specifically checking for the consumption of fiber‐rich foods by the patient. Utilizing both radiographic and endoscopic studies proves beneficial in pinpointing the location of the phytobezoar within the patient's alimentary tract. Timely surgical intervention is crucial to prevent the escalation of medical and surgical complications associated with small bowel obstruction.

## Author Contributions

**Aakash Pandit:** conceptualization, formal analysis, writing – original draft, writing – review and editing. **Melisha Koirala:** conceptualization, supervision, validation, writing – original draft, writing – review and editing. **Aashish Panta:** data curation, writing – original draft, writing – review and editing. **Nawadip Kandel:** data curation, writing – original draft, writing – review and editing.

## Funding

The authors have nothing to report.

## Disclosure

Other relationships: All authors have declared that there are no other relationships or activities that could appear to have influenced the submitted work.

## Consent

Written informed consent was obtained from the patient's parents/legal guardian for publication and any accompanying images. A copy of the written consent is available for review by the Editor‐in‐Chief of this journal on request.

## Conflicts of Interest

In compliance with the ICMJE uniform disclosure form, all authors declare the following:

## Data Availability

The data that support the findings of this study are available from the corresponding author upon reasonable request.
